# Commentary: A systematic review of the effectiveness of psychological interventions in organ transplantation

**DOI:** 10.3389/fpsyg.2026.1799549

**Published:** 2026-02-27

**Authors:** Kelly S. Clemens

**Affiliations:** Department of Psychology, Illinois State University, Normal, IL, United States

**Keywords:** biopsychosocial, context effects, nocebo, organ transplantation, placebo

## Introduction

The systematic review by De Pasquale et al.' (2025) is a substantial synthesis of the literature on psychological interventions in solid organ transplantation. By considering evidence across psychological treatment modalities, the authors demonstrate that psychological factors are relevant in transplantation and that psychological care can improve emotional wellbeing, treatment adherence, and quality of life.

At the same time, the review overlooks a dimension relevant to many reported outcomes: placebo and nocebo effects. Given the emotional and expectancy-rich nature of transplantation, omitting discussion of these effects may inadvertently fragment the literature and lead to missed opportunities to develop efficacious interventions. Indeed, explicit consideration of placebo and nocebo mechanisms does not undermine the value of psychological interventions but rather provides an explanatory mechanism for biopsychological interaction.

## Strengths of the review

De Pasquale et al.' (2025) emphasize the biopsychosocial nature of transplantation, noting that social factors such as identity reconstruction and psychological factors, including anxiety, guilt, treatment-relevant fear (i.e., fear of rejection), and depression, are associated with transplantation and potentially related to downstream outcomes, like medication adherence and quality of life. The authors also address the influence of psychological treatment on these factors and indicate that cognitive-behavioral therapy (CBT) and CBT-derived interventions demonstrated the strongest empirical support.

## A missing lens: placebo and nocebo effects

While there is a dearth of research on the influence of psychosocial factors on the physiological outcomes of transplantation (e.g., organ functionality; Sambucini et al., 2022), the confluence of cognitive, affective, and social factors described by the authors creates an environment for placebo and nocebo effects to thrive. Placebo effects refer to beneficial outcomes not caused by a treatment itself, while nocebo effects refer to non-beneficial outcomes (often treatment side effects) driven by negative expectations, fear, or anticipatory anxiety (Colloca and Barsky, 2020). Mechanisms for these effects include expectancy (Kirsch, 2018; Peerdeman et al., 2015; Sanders et al., 2020), conditioning (Babel, 2019, 2020), and affective (i.e., emotion-related) factors (Benedetti, 2014; Geers et al., 2020).

### Lessons from surgery, immunosuppressive treatment, and other types of transplantation

While often considered as medication effects, placebo effects are well-documented in surgical contexts. Sham-controlled trials have shown that invasive procedures can lead to improvements even when therapeutic elements are eliminated in procedures such as arthroscopic knee surgery (Moseley et al., 2002), vertebroplasty (Buchbinder et al., 2009), and other interventions.

Mechanisms influencing these effects may be amplified in transplantation. The emotional importance of receiving an organ and the intensity of perioperative care create an environment saturated with expectancy. Many outcomes reported in the review, including reductions in pain, anxiety, or distress following music therapy or mindfulness sessions, are consistent with well-established placebo-responsive domains.

There is limited evidence supporting placebo and nocebo effects in transplantation. Kirchhof et al. (2018) found when immunosuppressive drugs were paired with a gustatory stimulus to create a learned association, renal transplant patients who received the taste-immunosuppressive pairing demonstrated reduced T cell proliferative capacity compared to those who did not, suggesting amplification of immunosuppressive effects. Relatedly, in a study of patients with Parkinson's disease, participants were randomized to receive either a sham surgery or a transplantation of human embryonic dopamine neurons and were blinded to their condition. Participants who believed they had received the transplantation reported better outcomes, regardless of their actual condition (McRae et al., 2004).

These findings do not suggest that placebo effects can replace needed solid organ transplantation or immunosuppressive drugs, but rather suggest that psychosocial mechanisms may improve transplantation-relevant psychophysiological factors, which could be addressed in psychological treatments to potentially improve biopsychosocial functioning in adults after transplant. There is a need for future studies that examine the associations among psychological factors, psychological treatment, and physiological outcomes of transplantation are considered.

### Nocebo effects

Conditions for nocebo effects are also abundant in transplantation. The authors highlight the pervasiveness of psychological distress in many forms throughout their review, and psychological distress itself can function as a nocebo amplifier (Geers et al., 2020). This, along with well-intentioned but intensive patient education, may lead patients to expect complications, leading them to perceive benign sensations as problematic (e.g., signs of rejection). This phenomenon has been well-documented in pharmacological studies (e.g., Barsky, 2002), however remains unstudied in transplantation.

Several outcomes reviewed by De Pasquale et al.' (2025) (e.g., medication non-adherence, persistent anxiety despite successful transplantation) could plausibly reflect nocebo processes. Yet these effects are not addressed, leaving adverse outcomes individualized rather than contextualized. For example, while De Pasquale et al.' (2025) note that many patients must “cope with lifelong immunosuppressive therapies that can cause significant side effects—such as weight gain, tremors, or aesthetic changes—while contributing to anxiety, depression, and difficulties with body-image acceptance,” it is also possible that the experience of stress, anxiety, depression may lead to increased side effect experiences from immunosuppressive therapies.

There is, therefore, a need for focused research on nocebo effects in transplantation. Explicitly recognizing nocebo processes as relevant contributors to transplant outcomes would increase the issue's visibility and encourage future studies to incorporate expectancy, communication, and psychological context as integral components of transplant care to encourage biopsychosocial wellbeing.

## Discussion

De Pasquale et al.'s (2025) review represents a much-needed synthesis of how psychological intervention can improve psychosocial functioning and behaviors (e.g., treatment adherence) related to solid organ transplantation. Integrating placebo and nocebo mechanisms would deepen understanding of contextual factors shaping transplantation outcomes. Addressing placebo and nocebo effects may reframe the focus of interventions, allowing clinicians to harness beneficial expectations and emotions while mitigating harmful ones. Of note, recent development of *nocebo education* interventions offers an opportunity to integrate with existing cognitive-behavioral interventions to address how expectations, emotions, and attention to bodily sensations may influence the experience of nocebo effects (Spotts and Geers, 2025).

Transplantation is biologically and psychologically complex. De Pasquale et al.' (2025) note that “evidence indicates that emotional and psychological states directly affect treatment adherence, stress management, and, indirectly, clinical outcomes.” While this indirect relationship is strongly supported, integrating placebo and nocebo effects into our understanding of how psychosocial states impact transplantation can help us understand not only *indirect* clinical outcomes, but also *direct* clinical outcomes.
